# Glycine-Based [3+2] Cycloaddition for the Synthesis of Pyrrolidine-Containing Polycyclic Compounds

**DOI:** 10.3390/molecules29235726

**Published:** 2024-12-04

**Authors:** Tieli Zhou, Xiaofeng Zhang, Desheng Zhan, Wei Zhang

**Affiliations:** 1 College of Food Science and Engineering, Changchun University, Changchun 130022, China; zhoutieli00@sina.com; 2 Department of Cancer Biology, Dana-Farber Cancer Institute, Harvard University, Boston, MA 02215, USA; 3 Center for Green Chemistry, Department of Chemistry, University of Massachusetts Boston, 100 Morrissey Boulevard, Boston, MA 02125, USA; 4 Department of Chemistry, Changchun Normal University, Changchun 130031, China; zhandesheng@ccsfu.edu.cn

**Keywords:** Glycine, cycloaddition, pyrrolidine, azomethine ylides, annulation, dipolarophile

## Abstract

The synthesis of pyrrolidine compounds with biological interest is an active research topic. Glycine could be a versatile starting material for making pyrrolidine derivatives. This review covers recent works on glycine-based [3+2] cycloaddition and combines other annulation reactions in the one-pot synthesis of pyrrolidine-containing heterocyclic compounds. Synthetic method development, substrate scope, and reaction mechanisms are discussed. Applications of the compounds in drug discovery are briefly mentioned. This paper is helpful for chemists in the development of efficient and sustainable methods for the preparation of bioactive pyrrolidine compounds.

## 1. Introduction

**Figure 1:**
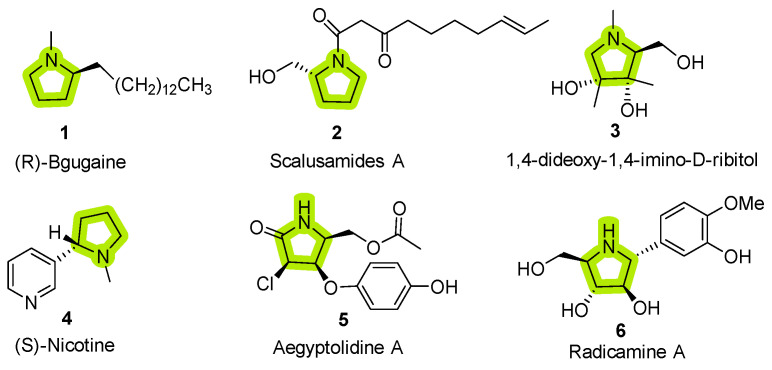
Representative pyrrolidine-containing natural products.

**Figure 2:**
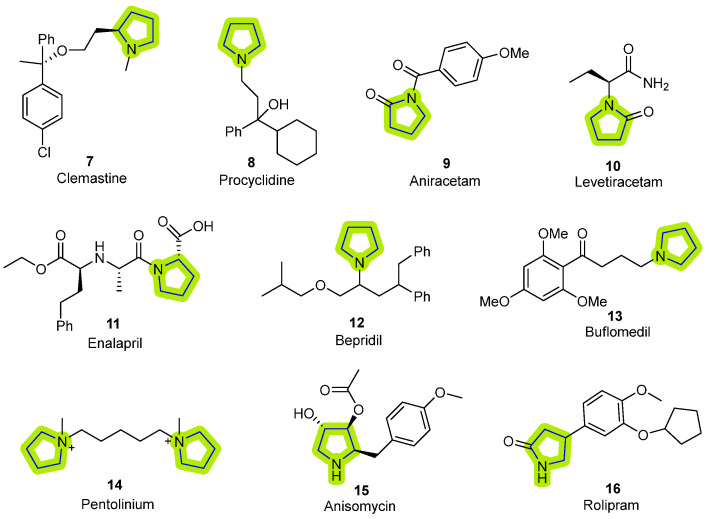
Representative FDA-approved pyrrolidine-containing drugs.

Nitrogen heterocycles are common motifs in natural products, bioactive compounds, and pharmaceutical chemicals [1,2]. Among them, five-membered secondary amine pyrrolidine (also known as tetrahydropyrrole) is a privileged scaffold **1**–**6** (Figure 1) [3,4,5,6]. In a drug development program, the introduction of the pyrrolidine ring into drug molecules could improve their water solubility and other physical and chemical properties [7]. Pyrrolidine increases the binding affinity of drug molecules and proteins due to the hydrogen bonding of pyrrolidine’s NH group. These features make pyrrolidine rings popular in the development of drug molecules such as US Food and Drug Administration (FDA)-approved drugs **7**–**16,** shown in Figure 2 [5,8,9,10,11,12,13].

**Scheme 1:**
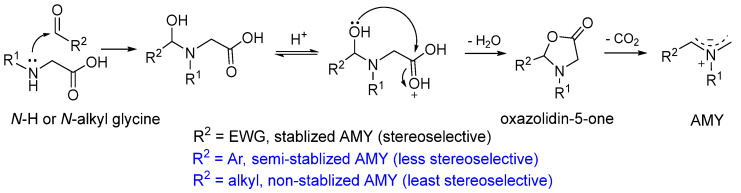
The formation of semi- and non-stabilized AMYs from glycines.

Over the years, the following general methods for the synthesis of pyrrolidine-containing compounds have been developed: (1) reductive amination involving the condensation of a 1,4-dicarbonyl compound with ammonia or a primary amine followed by reduction [14]; (2) the cyclization of amino alcohols or amino ketones, such as the cyclization of 1,4-amino alcohols under acidic or basic conditions [15]; and (3) the Mannich reaction, followed by cyclization and reduction [16]. Other than these methods, other approaches, such as organocatalysis [17], photoredox catalysis [18], intramolecular annulations [19], and [3+2] cycloadditions [20,21], have been developed, which have extended the scope for making pyrrolidines. This article highlights glycine-based decarboxylative 1,3-dipolar [3+2] cycloaddition for making pyrrolidine-containing heterocyclic compounds. Cycloaddition involves the generation of semi- or non-stabilized azomethine ylides (AMYs) via the decarboxylation of *N*-H or *N*-alkyl glycine-derived oxazolidine-5-ones. Semi-stabilized AMYs have better regio- and stereoselectivity than non-stabilized AMYs in cycloaddition (Scheme 1) [22]. Amino ester-based, derived AMYs require a base for deprotonation. While amino acid-derived AMYs need a caid for decarboxylation, the amino acid-based cycloaddition reaction can be combined with other annulation reactions for making bi-, tri-, and polycyclic systems. If the reaction involves an alkyl halide and generates HCl or HBr, then a base can be used as an acid scavenger to assist the reaction until completion [23].

## 2. Intermolecular [3+2] Cycloadditions

**Figure 3:**
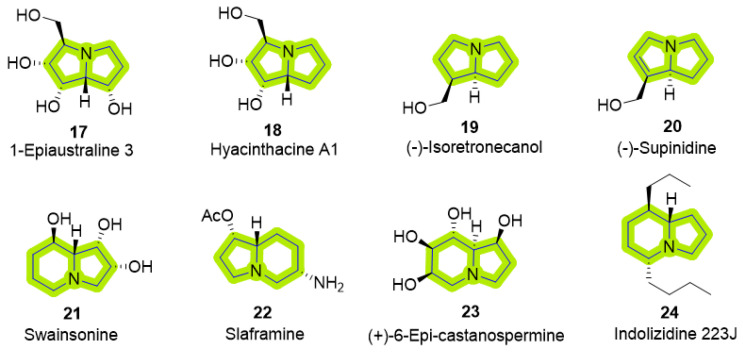
Pyrrolizidine- and indolizidine-bearing natural products.

Pyrrolizidine alkaloids have a bicyclic core of two five-membered rings sharing a fused nitrogen atom. Pyrrolizidine exists in natural products and drug molecules, with anti-inflammatory, antimicrobial, and anticancer activities **17**–**20** (Figure 3) [24,25,26]. Indolizidines consist of a bicyclic framework of five- and six-membered rings, sharing a fused nitrogen atom. The rigid yet versatile structure of indolizidine is a valuable scaffold in natural products and drug molecules as neurotransmitter modulators, enzyme inhibitors, and other therapeutic compounds **21**–**24** [27,28,29]. Both pyrrolizidines and indolizidines could serve as key intermediates in the synthesis of natural products and drug molecules. Their syntheses usually require multistep transformations, including cyclization and selective functionalization reactions [30,31,32,33,34,35]. Two cases for the synthesis of pyrrolizidine and indolizidine derivatives via cyclization and intermolecular [3+2] cycloaddition are presented in this section [36].

**Scheme 2:**
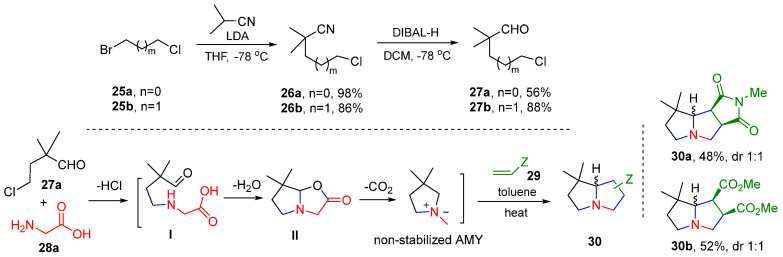
Synthesis of pyrrolizidines **30**.

**Scheme 3:**
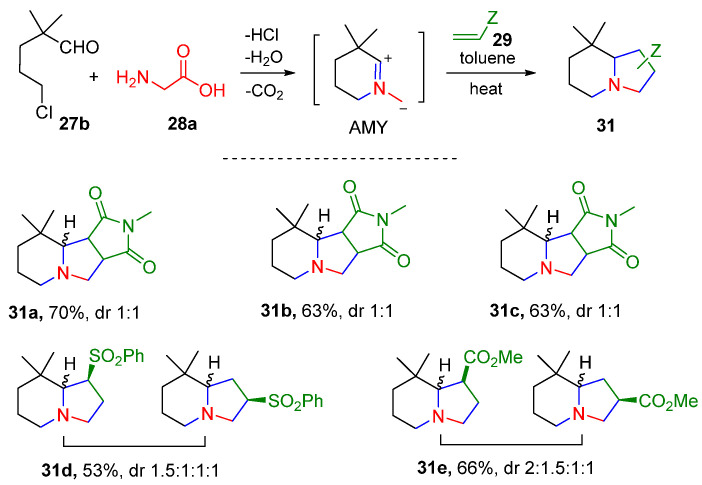
Synthesis of indolizidines **31**.

The Coldham group reported a cyclization and intermolecular [3+2] cycloaddition reaction process for the synthesis of pyrrolizidines and indolizidines [36]. The key intermediates **27a** and **27b** were prepared in two steps, involving the alkylation of 1-bromo-2-chloroethane or 1-bromo-3-chloropropane with isobutyronitrile to give **26a** and **26b,** followed by the reduction of the CN group with DIBAL-H (Scheme 2). AMY generated from the reaction of aldehyde **27a** or **27b** with glycine **28a** underwent 1,3-dipolar cycloaddition with dipolarophiles **29** to afford pyrrolizidines **30a**–**b** in a 48% and 52% yield, or indolizidines **31a**–**e** in a 53–70% yield, with low dr 1:1 due to non-stabilized AMY (Scheme 2 and Scheme 3).

**Scheme 4:**
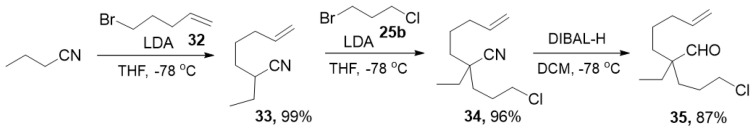
Preparation of aldehyde **35**.

**Scheme 5:**
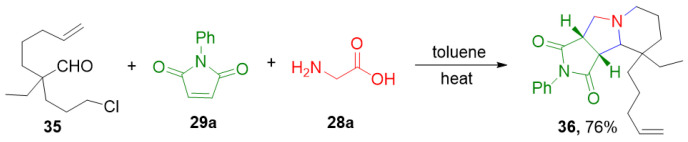
Intermolecular cycloaddition for indolizidine **36**.

The Burrell group reported the intermolecular 1,3-dipolar cycloaddition of aldehyde **35** for the construction of congregated triheterocycle **36.** The alkylation of butanenitrile with 5-bromo-1-pentene **32** afforded **33**, which underwent another alkylation with 1-bromo-4-chlorobutane **25b** to afford **34**, followed by DIBAL-H reduction, to give aldehyde **35** (Scheme 4) [37]. Heating **35** with glycine **28a** and dipolarophile *N*-phenylmaleimide **29a** gave a mixture of stereoisomers of indolizidine **36** as an example of non-stabilized AMY (Scheme 5).

## 3. Intramolecular Cycloadditions

**Figure 4:**
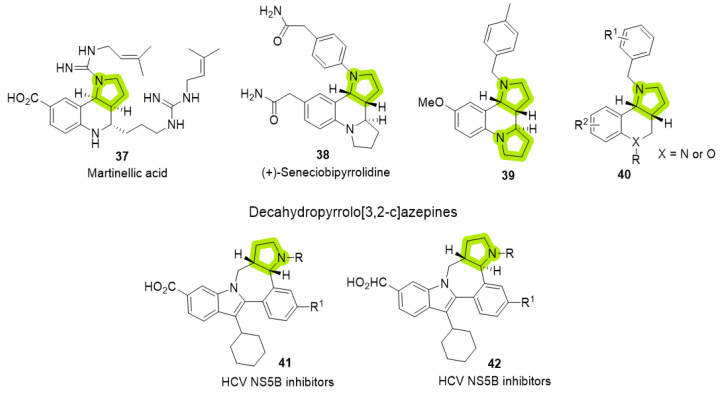
Bioactive compounds containing pyrrolidine.

Octahydro-1*H*-pyrrolo[3,2-*c*]pyridines and octahydropyrano[4,3-*b*]pyrroles consist of a fused bicyclic six-membered and five-membered ring with nitrogen or oxygen atoms. They can be found in drug molecules, such as agents for central nervous system (CNS) disorders, anti-infective agents, and anticancer drug candidates [38,39]. For example, martinellic acid **37** and (±)-seneciobipyrrolidine **38** (Figure 4) are natural alkaloids which can interact with different biological targets for diverse therapeutic utilities [40,41,42]. Dong group discovered that compounds **39** and **40** could upregulate eukaryotic translation initiation factor 4E-binding protein 1 (4E-BP1) to enhance the Akt and AMP-activated protein kinase (AMPK) phosphorylation, thereby promoting glucose uptake [42]. Habermann‘s lab reported pentacyclic decahydropyrrolo[3,2-*c*]azepines **41** and **42** as hepatitis C virus (HCV) NS5B polymerase inhibitors [43]. The fused bicyclic scaffolds **40**–**42** could be synthesized by glycine-based intramolecular cycloadditions.

**Scheme 6:**
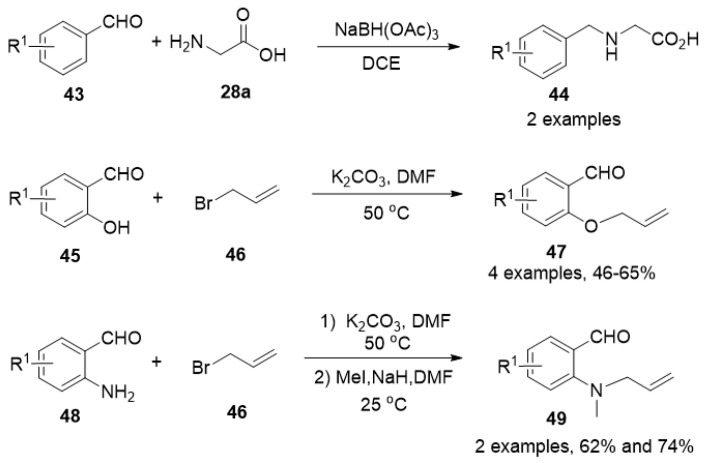
Preparation of intermediates **44**, **47**, and **49**.

**Scheme 7:**
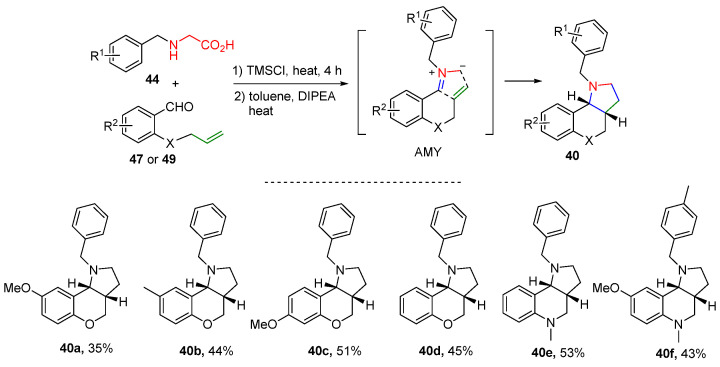
Synthesis of octahydro-1*H*-pyrrolo[3,2-*c*]pyridines and octahydropyrano[4,3-*b*]pyrroles.

To evaluate the impact of octahydro-1*H*-pyrrolo[3,2-*c*]pyridines and octahydropyrano[4,3-*b*]pyrroles on glucose uptake, the Dong group made intermediate **44** by the reductive amination of glycine and aldehydes **43**, as well as intermediates **47** and **49** by the alkylation of aldehydes **45** and **48** with 3-bromopropylene **46** (Scheme 6) [42]. The authors then performed double annulations of these three intermediates by decarboxylative intramolecular [3+2] cycloaddition to obtain compounds **40a**–**f** in 35–53% yields (Scheme 7). Compound **40f** has an 80.6% glucose uptake, but the uptake activation was lower after changing the substituents on the aryl ring of octahydropyrano[4,3-*b*]pyrroles **40a**–**d** and octahydro-1*H*-pyrrolo[3,2-*c*]pyridines **40e**.

**Figure 5:**
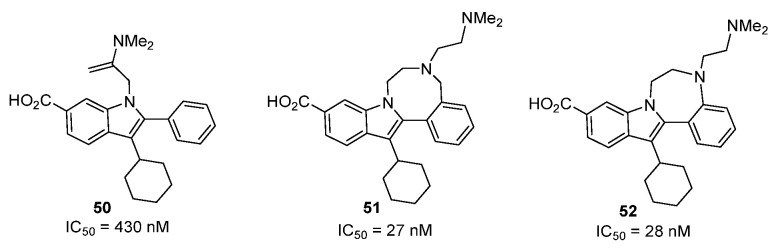
Examples of HCV NS5B inhibitors.

Decahydropyrrolo[3,2-*c*]azepines, such as **52**, have a seven-membered azepine ring fused to an indole moiety to form a rigid and well-defined three-dimensional structure. This scaffold has been introduced in drug molecules for treating neurological disorders and used as an anticancer and antimicrobial agent. The Habermann group initially reported benzimidazole **50** and then tetracyclic indole carboxylic acid derivatives **51** and **52** as potent and high-selectivity inhibitors for HCV NS5B polymerase (Figure 5) [44,45,46]. The inclusion of a cyclohexyl ring and an aromatic ring bonded to a heterocyclic core is a consistent and defining characteristic of these compounds. Studies on the tetracyclic indole series indicated the positive impact of an exposed basic functionality by decreasing the rotatable bond count via the incorporation of a side chain into the rigid ring system [43].

**Scheme 8:**
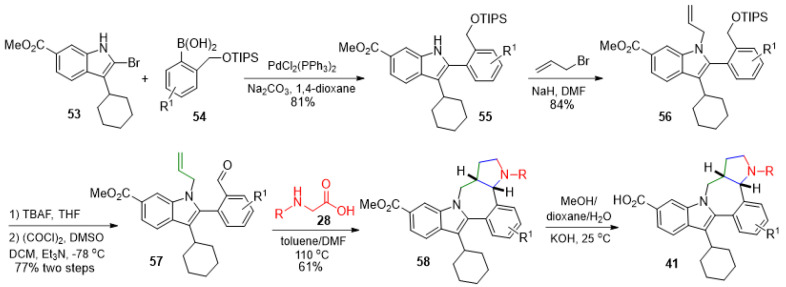
The synthesis of cis-pentacyclic compounds **41**.

**Figure 6:**
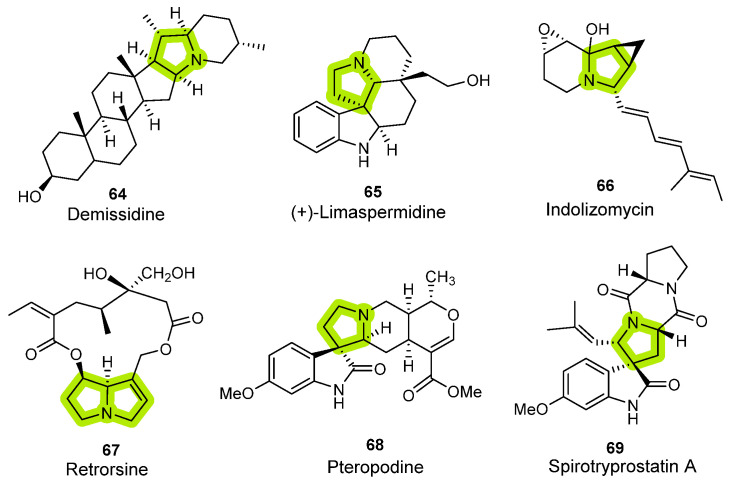
Pyrrolidine-containing fused and spiro natural products.

Habermann’s group developed synthetic routes for the synthesis of both *cis*-pentacyclic compounds **41** and *trans*-pentacyclic compounds **42** by conducting the intramolecular [3+2] cycloaddition of glycine to form the pyrrolidine ring [43]. As shown in Scheme 8, intermediates **55** were generated by the Suzuki coupling of compounds **53** and **54**, followed by *N*-allylation with allyl bromide to form intermediates **56**. After deprotection and the Swern oxidation of **56**, compounds **57** were used for intramolecular [3+2] cycloaddition with glycine or *N*-substituted glycine **28** to form *cis*-pentacyclic system **58**, and then products **41a**–**b** after hydrolysis of the ester group (Scheme 8); the *cis*-pentacyclic stereochemistry was confirmed by the reported intramolecular 1,3-dipolar cycloadditions of the same kind [47]. They found both compounds are good allosteric inhibitors of HCV NS5B polymerase with 31 and 21 nM of IC_50_ via cell-based assay (Figure 6).

**Scheme 9:**
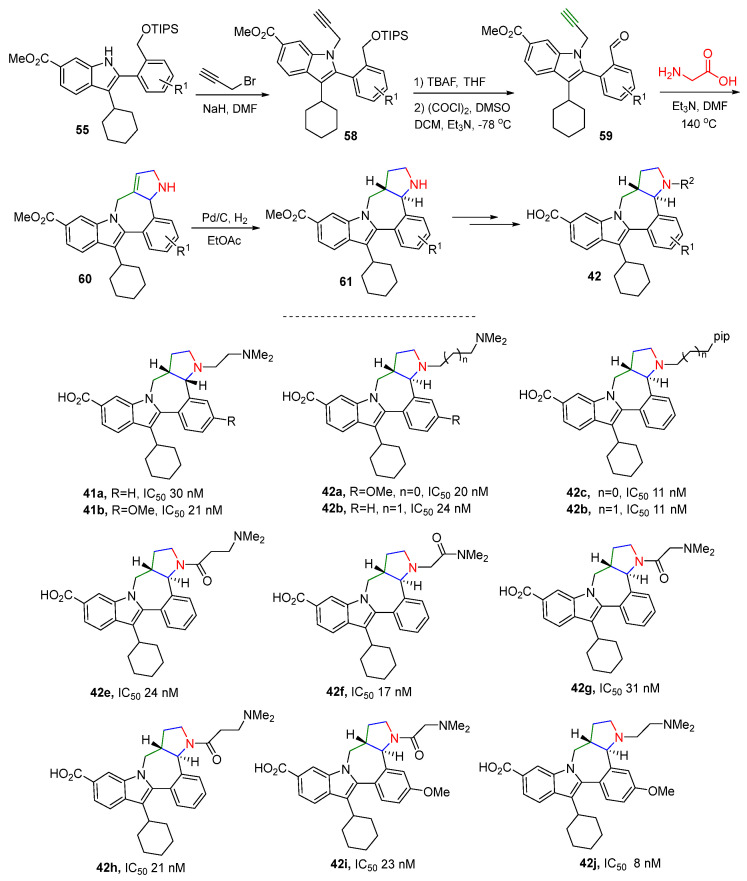
Synthesis of *trans*-pentacyclic compounds **42** and their bioactivities.

**Scheme 10:**
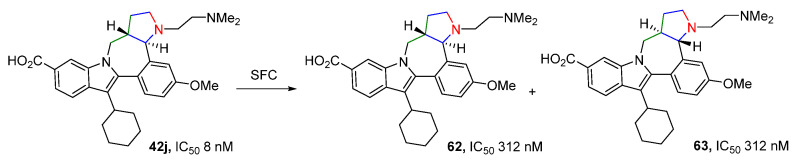
Two enantiomers of compound **42j** and their bioactivities.

A similar synthetic route using propargyl bromide instead of allyl bromide for the *N*-propargylation of **55** to form **58**, followed by deprotection and the Swern oxidation, gave aldehydes **59.** The intramolecular [3+2] cycloaddition of **59** with glycine to generate compound **60**, and sequential hydrogenation over palladium led to *trans*-pentacyclic **61**, followed by other transformations, could lead to the formation of *trans*-pentacyclic compounds **42a**–**j** (Scheme 9). They are validated inhibitors of HCV NS5B polymerase in the range of 8–31 nM via cellular assay [43]. Among them, *trans*-pentacyclic compound **42j** shows the most potency with 8 nM of IC_50_. The SFC separation of **42j** gave two, enantiomers **62** and **63**, which have IC_50_ 312 and 4 nM, respectively (Scheme 10). A model study indicated that **63** could efficiently block subgenomic HCV RNA replication in the cell line at low nanomolar concentrations, which makes it seven-fold more potent than tetracyclic compound **52.**

## 4. Double Intermolecular [3+2] Cycloadditions

Pyrrolidine-containing polyheterocyclic alkaloids have complex structures and diverse biological activities. Shown in Figure 6 are some polyheterocyclic natural products **64**–**69** containing pyrrolizidine, indolizidine, or spiro-oxindole rings [48,49,50,51,52]. These compounds could have pharmacological activities which make them attractive in the development of new drugs. The incorporation of pyrrolidine in polycyclic frameworks contributes to the structure diversity and complexity of these molecules, which could enhance their binding affinity and specificity to biological targets. Their ability to interact with a variety of biological targets, including enzymes, receptors, and nucleic acids, further underscores their importance in medicinal chemistry.

### 4.1. Double Cycloadditions for Tetracyclic Pyrrolizidines

**Scheme 11:**
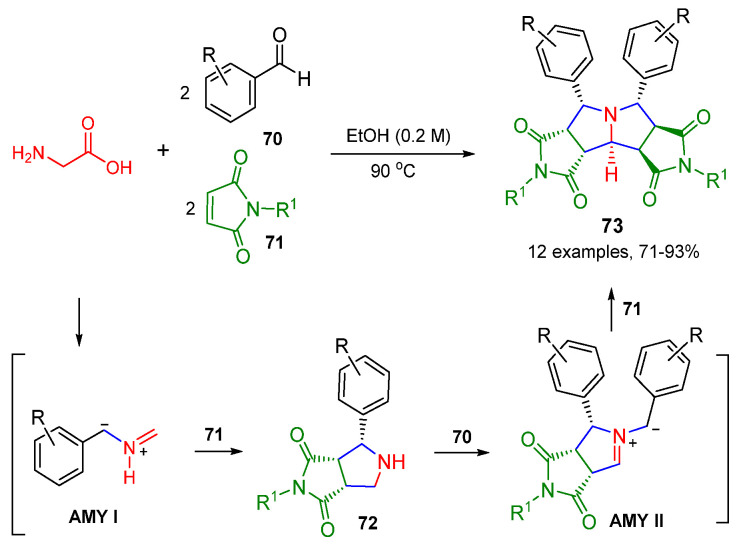
Pseudo five-component synthesis of tetracyclic pyrrolizidines **73**.

**Scheme 12:**
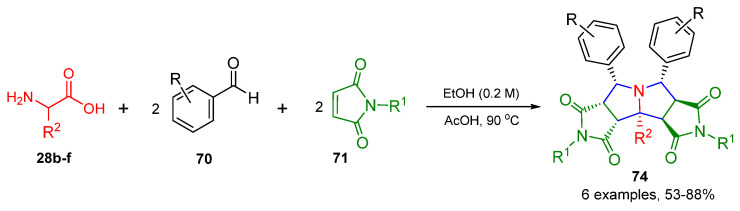
Synthesis of tetracyclic pyrrolizidines **74**.

A highly efficient decarboxylative double [3+2] cycloaddition reaction for the synthesis of tetracyclic pyrrolizidines was reported by Zhang and co-workers [53]. The reaction process involves the formation of two semi-stabilized azomethine ylides derived from the decarboxylation of amino acids and the α-C-H functionalization of newly formed pyrrolidines. It is a pseudo five-component reaction (A + 2B + 2C) of glycine (1 equiv.), aldehydes **70** (2 equiv.), and maleimides **71** (2 equiv.) to create tetracyclic pyrrolizidines **73** in 71–93% yields with > 9:1 dr (Scheme 11). In addition, using α-substituted glycines **28b**–**f** (R^2^ = Me, Et, CH_2_OH, *^i^*Bu, and Pr), this synthetic approach could give diverse tetracyclic pyrrolizidines **74** in 53–88% yields with dr > 9:1 (Scheme 12). The stereochemistry of **73** and **74** was determined by single-crystal X-ray diffraction and NMR analysis.

**Scheme 13:**
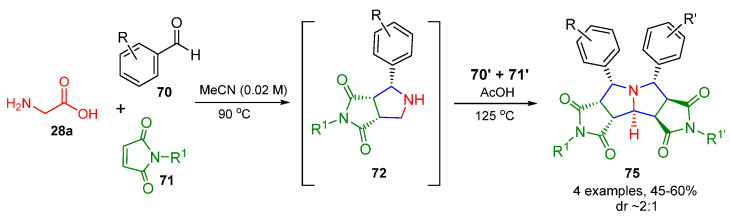
One-pot and two-step synthesis of tetracyclic pyrrolizidines **75**.

Different from the pseudo five-component reactions (A + 2B + 2C) presented in Scheme 11 and Scheme 12, one-pot two-step reactions (A + B + C + B′ + C′) could introduce a different set of B′ and C′ for the second cycloaddition (Scheme 13) [53]. The first cycloaddition of glycine with aldehydes **70** and maleimides **71** for making adducts **72** were conducted at a low concentration of glycine in MeCN (0.02 M) at 90 °C for 6 h. A new set of aldehydes 70′ and maleimides 71′ were introduced for the second cycloaddition at 125 °C for 12 h under the catalysis of acetic acid to generate tetracyclic pyrrolizidines **75** in 45–60% yields, but with a lower dr of ~2:1 (Scheme 13). Additionally, regarding the significant differences between five component reactions (A + 2B + 2C) and one-pot two-step processes (A + B + C + B′ + C′), the authors clearly identified and characterized compounds **73** and **74** by single-crystal X-ray diffraction and NMR spectroscopy. However, compound **75** exhibited many uncertainties due to the low-purity of **75**, which requires further analysis to identify stereochemistry in the future [53].

### 4.2. Double Cycloaddition for Bispiro[Oxindole-Pyrrolidine]s

**Scheme 14:**
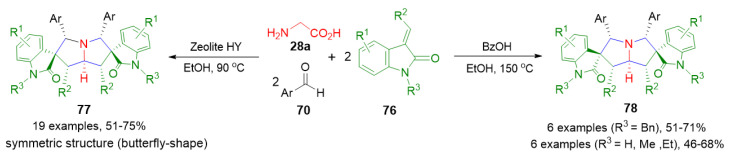
Double cycloaddition involving glycine and olefinic oxindoles.

**Scheme 15:**
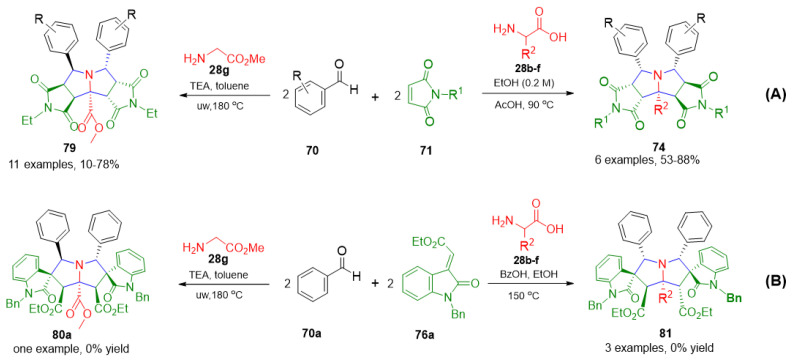
Comparison of maleimides and olefinic oxindoles in double cycloadditions.

As part of a continuous effort in the development of A + 2B + 2C-type double [3+2] cycloadditions for the construction of pyrrolizidines, Zhang and co-workers synthesized dispirooxindole-pyrrolizidines **77**, which have a butterfly-shaped structure with a plane of symmetry. The three-dimensional pore structure of zeolite HY has a regulating effect on stereoselectivity. Under the heterogeneous catalysis of zeolite HY, the reaction of glycine, aldehydes **70**, and olefinic oxindoles **76** at 90 °C for 6 h gave double cyclization products **77** in 51–75% yields (Scheme 14) [54]. It was found that the presence of R^3^ (Bn) on compound **76** could generate alternative diastereomers **78** as the major products in a 51–71% yield. This reaction is thermodynamically favored at 150 °C under the catalysis of BzOH. The reactions of olefinic oxindoles **76** with R^3^ as H, Me, or Et s could give products **78**, but with a lower dr than that of R^3^ is Bn (Scheme 14). It was found that maleimides **71** are good dipolarophiles in the (A + 2B + 2C)-kind double cycloaddition reactions of glycine ester hydrochloride **28g** or α-substituted glycines **28b**–**f** for synthesizing products **79** and **74** (Scheme 15, (A)) [54]. Different to the decarboxylative [3+2] cycloaddition of glycine, the preparation of **79** was accomplished through stabilized AMY-based [3+2] cycloaddition via the deprotonation of glycine ester 28g reacting with aldehydes. TEA is used as a base for the deprotonative formation of the AMY. In contrast, double cycloaddition using **76a** as a dipolarophile could not give the expected products **80a** and **81** (Scheme 15, (B)).

## 5. Cyclization and Intramolecular Cycloaddition Reactions

**Figure 7:**
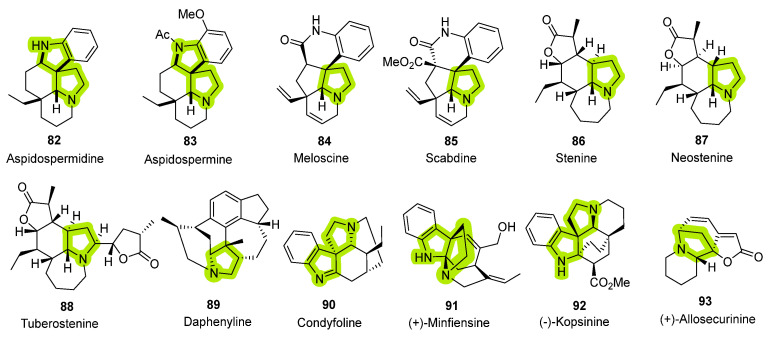
Pyrrolidine-containing congregated polycyclic natural products.

Pyrrolidine-containing congested polycycles can be found in natural products, such as **82**–**93**, which have a broad spectrum of biological activities (Figure 7) [55]. In drug discovery, the incorporation of pyrrolidine into polycycles introduces conformational constraint and steric hindrance, which could modulate the pharmacokinetic and pharmacodynamic properties of drug molecules. The synthesis of pyrrolidine-containing polycycles is a challenging and rewarding endeavor. Recent advances in transition-metal-catalyzed reactions, cycloaddition reactions, and multicomponent reactions have enabled the synthesis of these complex structures. Presented in this section is glycine-based cyclization followed by intramolecular cycloaddition reactions to construct pyrrolidine-containing congested scaffolds which can be found in aspidosperma, meloscine, scandine, stemona, and daphniphyllum alkaloids.

### 5.1. Synthesis of Aspidosperma Alkaloids

**Scheme 16:**
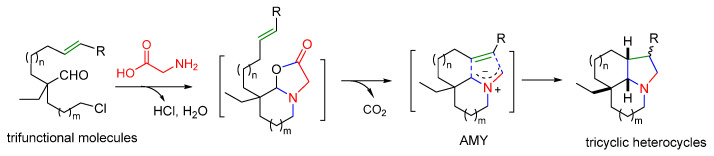
Cyclization and cycloaddition cascade for constructing tricyclic amines.

Combining cyclization with cycloaddition as a cascade reaction is a good strategy for efficiently synthesizing polycyclic scaffolds. For instance, the decarboxylative cyclization of glycine with an aldehyde group to generate AMY for sequential intramolecular cycloaddition is shown in Scheme 16 [55]. The Coldham group developed a cascade process to make tricyclic amines from acyclic precursors in the total synthesis of natural alkaloids, such **82** and **83** (Figure 3).

**Scheme 17:**
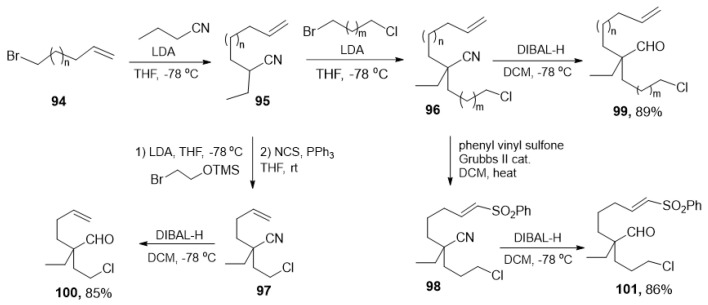
Preparation of multi-functional aldehydes **99**–**101**.

**Scheme 18:**
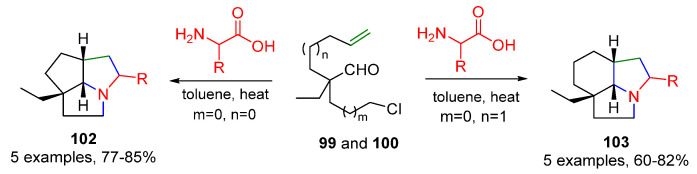
The synthesis of tricyclic amines **102** and **103**.

**Scheme 19:**
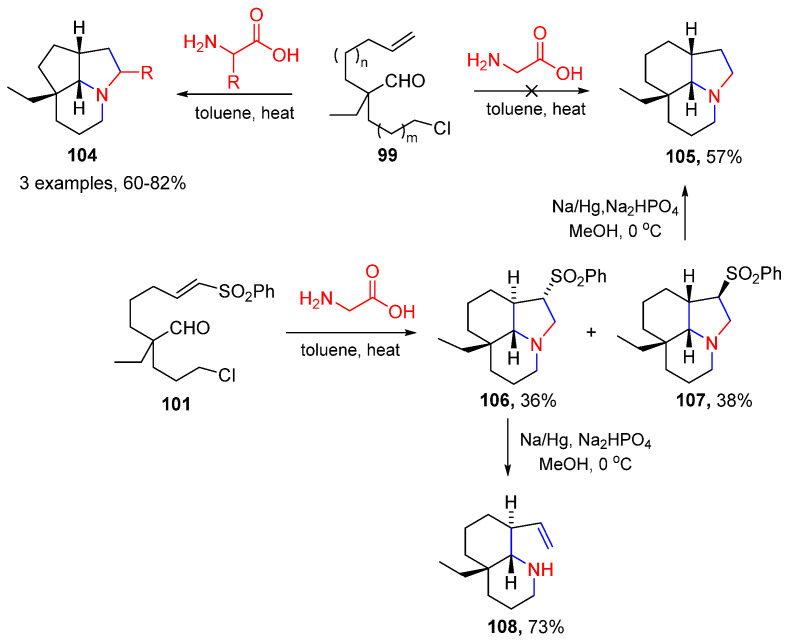
Synthesis of tricyclic amines **104** and **105**.

The key for the cascade synthesis of tricyclic amines involving cycloaddition is to form a cyclic azomethine ylide from aldehyde and primary amine, followed by in situ cyclative *N*-alkylation. The reactions need multi-functional aldehydes **99**–**101** to trigger the cascade reaction process. The Coldham group reported the synthesis of aldehydes **99**–**100** with a 85–89% yield by dialkylations of butanenitrile with bromo-substrates **94** to make intermediates **96** and **97**. The reduction of nitrile of **96** or **97** afforded **99** and **100**, respectively, while the cross-metathesis of **96** with phenyl vinyl sulfone afforded **98**, followed by reduction, to form **101** (Scheme 17) [37,56]. With key substrates **99** and **100** in hand, the synthesis of tricyclic amines **102** and **103** via cascade annulations with α-substituted glycines was accomplished (Scheme 18). Applications of the method for the synthesis of tricyclic amines **104** with aldehydes **99** was successful, but this was not the case for the preparation of **105** due to nonactivated alkene (Scheme 19). The reaction of **101** with glycine afforded tricyclic amines **106** and **107** by cyclization/intramolecular cycloaddition. The sulfonyl group in **107** was removed using a sodium/mercury amalgam to give amine **105**, whereas the desulfonylation of **106** afforded bicyclic product **108** via *β*-elimination (Scheme 19).

**Scheme 20:**
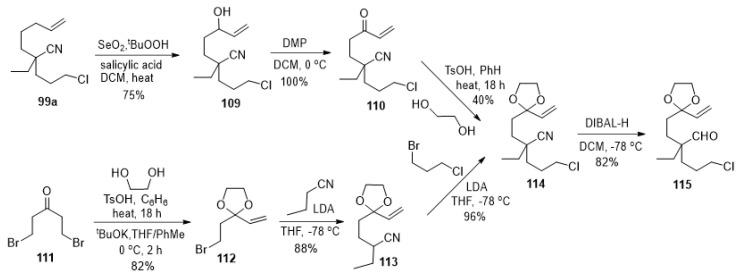
Preparation of multi-functional compound **115**.

After completing the synthesis of tricyclic amines **104**–**107** by annulations of trifunctional substrates with glycine, shown in Scheme 18 and Scheme 19, the Coldham group applied the method in the preparation of aspidosperma alkaloids [37,56]. Two pathways for making trifunctional substrate **115** were proposed in Scheme 20. The first one involved the allylic oxidation of compound **99a** for making alcohol **109**, followed by oxidation with Dess-Martin periodinane reagent and the protection of ketone **110** with ethylene glycol to form compound **114**. The second one started from the protection of ketone **111** with ethylene glycol to form **112**, which was then used for the alkylation of butanenitrile to afford compound **113**. Another alkylation with 1-bromo-3-chloropropane gave **114** (Scheme 20). Trifunctional compound **115** was made by the reduction of the nitrile group with DIBAL-H.

**Scheme 21:**
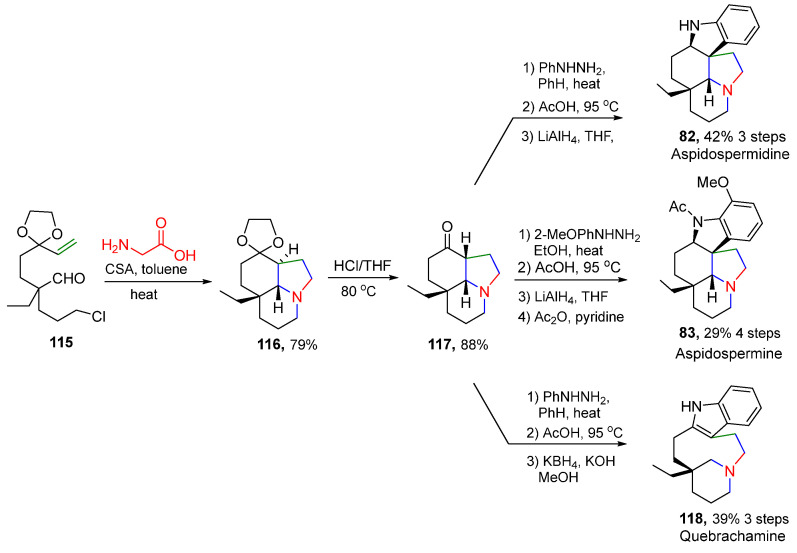
Synthesis of aspidosperma alkaloids **82**, **83**, and **118**.

The annulation of aldehyde **115** with glycine was successfully carried out for the synthesis of tricyclic amine **116** (Scheme 21) [37,56]. The hydrolysis of the acetal group of **116** gave *cis/cis* ketone **117**, involving the epimerization of the α-proton of the carbonyl. Ketone **117** was used for the synthesis of alkaloids aspidospermidine **82**, aspidospermine **83**, and quebrachamine. The treatment of **117** with phenylhydrazine in benzene, and then in acetic acid, followed by the removal of solvent and heating with LiAlH_4_ in THF, afforded (±)-aspidospermidine **82** in a 42% yield over three steps. Similarly, heating ketone **117** with 2-methoxyphenylhydrazine in EtOH generated the intermediate hydrazone that was heated in acetic acid. After partial purification via a short silica column, the crude mixture was dissolved in THF and treated with LiAlH_4_, and then acetylation, to afford alkaloid (±)-aspidospermine **83** in a 29% yield over four steps. In addition, the reaction of ketone 117 with phenylhydrazine in benzene then in acetic acid, followed by the removal of solvent and heating with KBH_4_ in methanolic KOH, gave (±)-quebrachamine **118** in a 39% yield over three steps.

### 5.2. Synthesis Tricyclic Core of Meloscine and Scandine

**Scheme 22:**
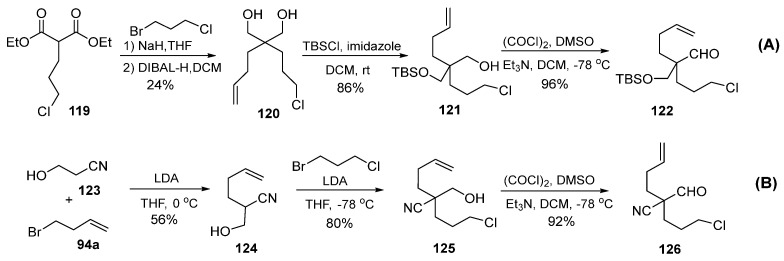
Preparation of multi-functional aldehydes **122** (**A**) and **126** (**B**).

The Coldham group applied the cascade annulation method in the construction of the tricyclic core of meloscine **84** and scandine **85** [57]. Key intermediates **122** and **126** were used following the protocols mentioned in the previous section (Scheme 22). The alkylation of the substituted malonate **119** followed by the reduction of both esters with DIBAL-H gave diol **120**. The mono-protection of **120** with TBSCl afforded alcohol **121**, followed by the Swern oxidation, to give aldehyde **122**. The dialkylation of nitrile **123** with **94a**, and then with 1-bromo-3-chloropropane, occurred smoothly to give compound **125**, and then **126** after Swern oxidation.

**Scheme 23:**
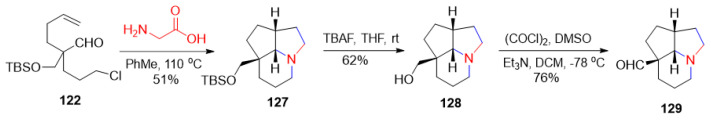
The synthesis of tricyclic amine **129**.

**Scheme 24:**
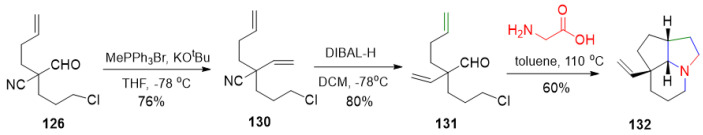
The synthesis of tricyclic amine **132**.

Following the general procedures for cascade annulation, aldehyde **122** reacted with glycine in toluene under heating gave intermediate **127**, followed by the deprotection of the TBS group and the Swern oxidation, to afford tricyclic amines **129** (Scheme 23). Another trifunctional substrate **131** was made by the olefination of aldehyde **126** and the nitrile reduction of **130**. Heating **131** with glycine afforded tricyclic compound **132,** which is the core of meloscine **84** and scandine **85** (Scheme 24) [57].

### 5.3. Synthesis Tricyclic Core of Stemona Alkaloids

**Scheme 25:**
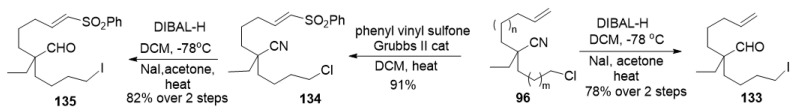
Preparation of multi-functional aldehydes **133** and **135**.

**Scheme 26:**
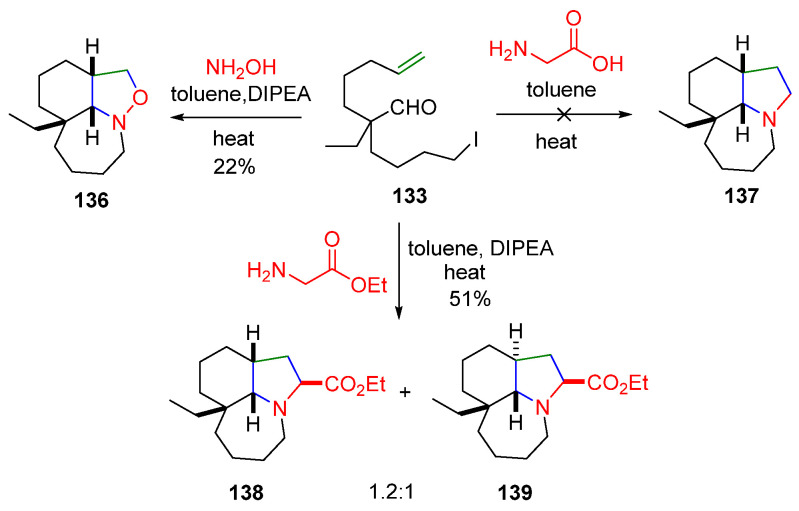
Synthesis of tricyclic amines **136**, **138**, and **139**.

**Scheme 27:**
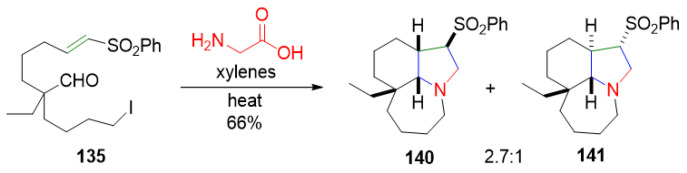
Synthesis of tricyclic amines **140** and **141**.

The Coldham group applied a similar method in the synthesize of the core ring of stemona alkaloids **86**–**88**. Compound **96,** shown in Scheme 17, was used to make modified trifunctional compounds **133** and **135** (Scheme 25) [58]. Compound **133** derived from the cyano group reduction of **96**, whereas **135** was made by metathesis of 96 with phenyl vinyl sulfone for 134, followed by cyano group reduction. The treatment of aldehyde **133** with hydroxylamine hydrochloride under heating generated compound **136** as a single diastereoisomer in a low yield of 22%. The reaction of aldehyde **133** and glycine did not give **137**. However, the reaction of glycine ethyl ester with aldehyde **133** generated stabilized azomethine ylide for cycloaddition to give cycloadducts **138** and **139** in 1.2:1 dr (Scheme 26). The stereochemistry of tricyclic compounds **138** and **139** were determined by the crystal X-ray analysis of reduced esters. In addition, the treatment of aldehyde **135** with glycine in xylene gave cycloadducts **140** and **141** in 66% yield with dr = 2.7:1 (Scheme 27) [58].

### 5.4. Synthesis of Bridged Tricyclic Core of Daphniphyllum Alkaloids

**Scheme 28:**
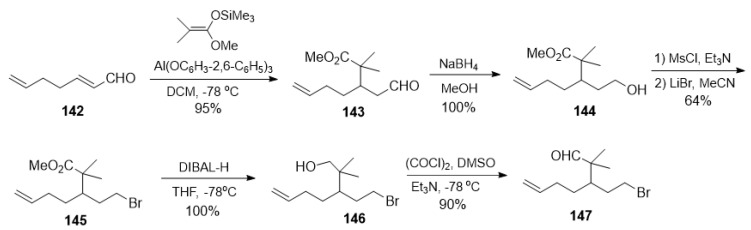
Preparation of multi-functional aldehyde **147**.

**Scheme 29:**
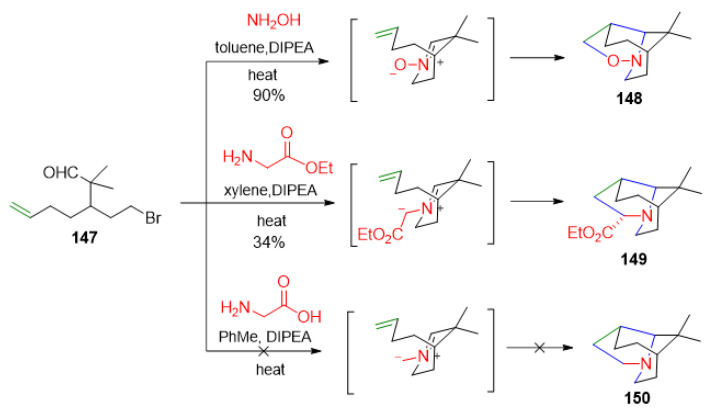
The synthesis of bridged tricyclic amines **148** and **149**.

**Scheme 30:**
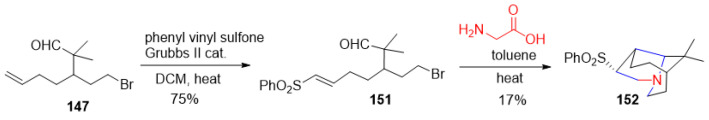
The synthesis of bridged tricyclic amines **152**.

In their continuous effort to develop a new method for making tricyclic amines, the Coldham group designed a new trifunctional compound **147** which could be used to form a bridged tricyclic core. The conjugate addition of aldehyde **142** with a silyl ketene acetal gave **143**, followed by the reduction and alcohol mesylation of **144** for MsO/Br exchange, to give compound **145**. The reduction of **145** and Swern oxidation afforded **147** (Scheme 28) [59]. Compound **147** was used for cascade annulation for the construction of bridged tricyclic amines **148** and **149**, which are a core structure in daphniphyllum alkaloids. The reaction of **147** with hydroxylamine hydrochloride or glycine ethyl ester afforded nitrone or azomethine ylides for intramolecular [3+2] cycloaddition to form **148** or **149**. However, the reaction of aldehyde **147** and glycine failed to give pyrrolidine product **150** (Scheme 29) [59]. To increase the reactivity of azomethine ylide for cycloaddition, an electron-withdrawing group was introduced to alkene **151** by the cross metathesis of **147** with phenyl vinyl sulfone (Scheme 30). The activated alkene **151** led to the formation of stabilized azomethine ylide for cycloaddition to form **152**, but still in a low yield of 17%.

## 6. Conclusions

Glycine-based decarboxylative 1,3-dipolar [3+2] cycloaddition integrated with other annulation reactions provides powerful approaches for the synthesis of pyrrolidine-containing polycycles. This review highlights the versatility of glycine as a basic building block in the synthesis of complex molecules, particularly the pyrrolidine framework. This approach has the advantages of having few synthetic steps, minimal byproduct formation, high atom economy, and good synthetic efficiency. These methods could be attractive to both academic studies and industrial applications. As the research on this topic continues to evolve, glycine-based annulation reactions are expected to play an increasingly important role in the development of novel and complex structures related to natural products and drug molecules.
